# Supplementary material for: A choreography of centrosomal mRNAs reveals a conserved localization mechanism involving active polysome transport
Source: Nat Commun. 2021 Mar 1;12:1352. doi: 10.1038/s41467-021-21585-7 (PMC7921559; doi:10.1038/s41467-021-21585-7)
Supplement: Supplementary file 1 — Supplemetary Information [file 41467_2021_21585_MOESM1_ESM.pdf]

A choreography of centrosomal mRNAs reveals a conserved localization mechanism involving active polysome transport

Adham Safieddine, Emeline Coleno, Soha Salloum, Arthur Imbert, Abdel-Meneem Traboulsi, Oh Sung Kwon, Frederic Lionneton, Virginie Georget, Marie-Cécile Robert, Thierry Gostan, Charles-Henri Lecellier, Racha Chouaib, Xavier Pichon, Hervé Le Hir, Kazem Zibara, Florian Müller, Thomas Walter, Marion Peter, Edouard Bertrand

Supplementary Figures and table

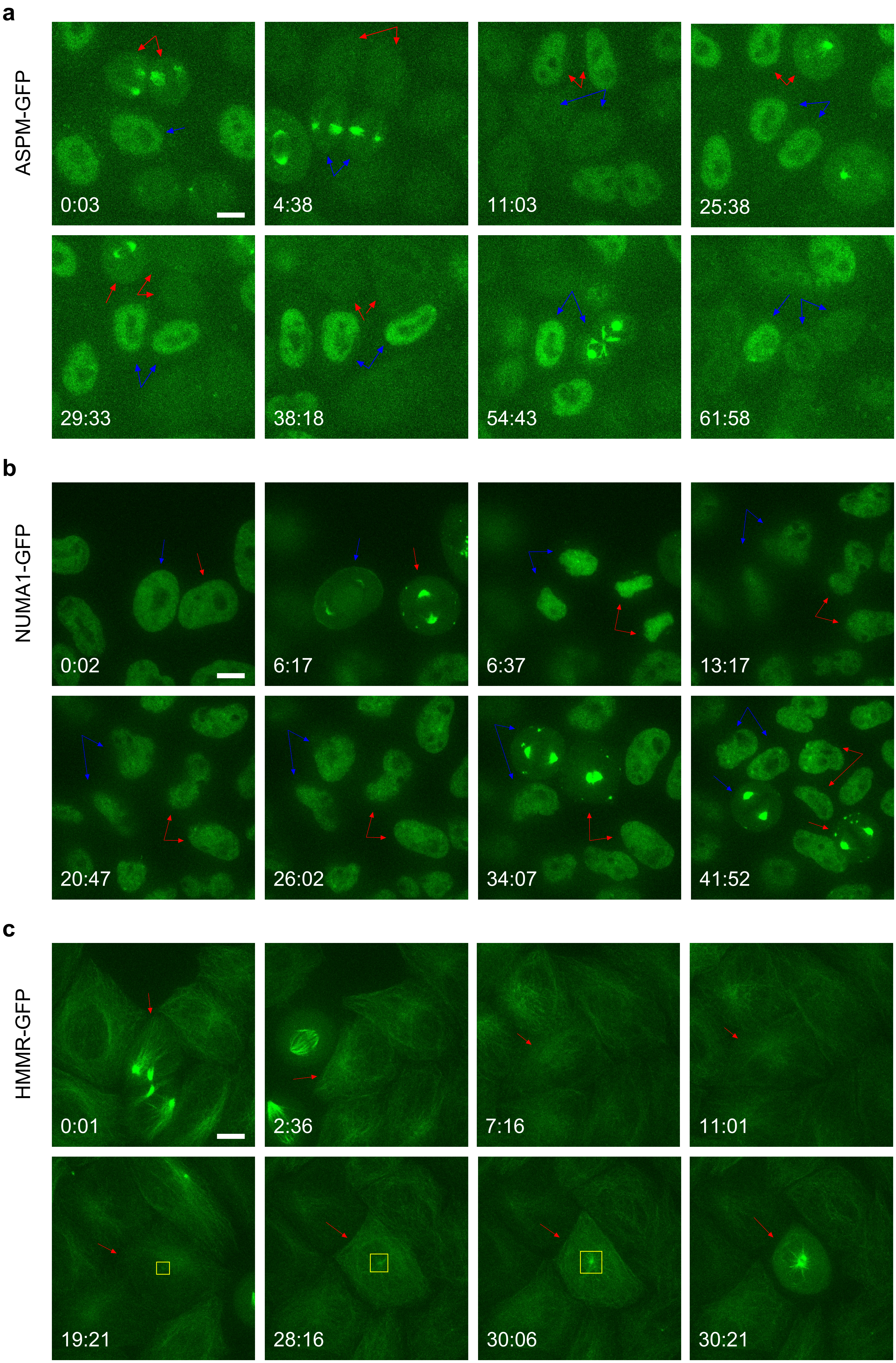
a
ASPM-GFP
0:03
4:38
11:03
25:38
29:33
38:18
54:43
61:58
b
NUMA1-GFP
0:02
6:17
6:37
13:17
20:47
26:02
34:07
41:52
c
HMMR-GFP
0:01
2:36
7:16
11:01
19:21
28:16
30:06
30:21

Figure S1

Supplementary Fig. 1 (related to Fig. 2): Expression and localization of ASPM, NUMA1, and HMMR proteins across an entire cell cycle.

a Images are snapshots of living HeLa cells expressing an ASPM-GFP BAC and imaged across an entire cell cycle. Signal is in green and corresponds to the ASPM-GFP protein. Scale bar is 10 microns and time is in hours: minutes. Red and blue arrows follow two dividing cells. Experiment repeated three times with similar outcomes.

b Legend as in **a**, but for HeLa cells containing a NUMA1-GFP BAC.

c Legend as in **a**, but for HeLa cells containing a HMMR-GFP BAC.

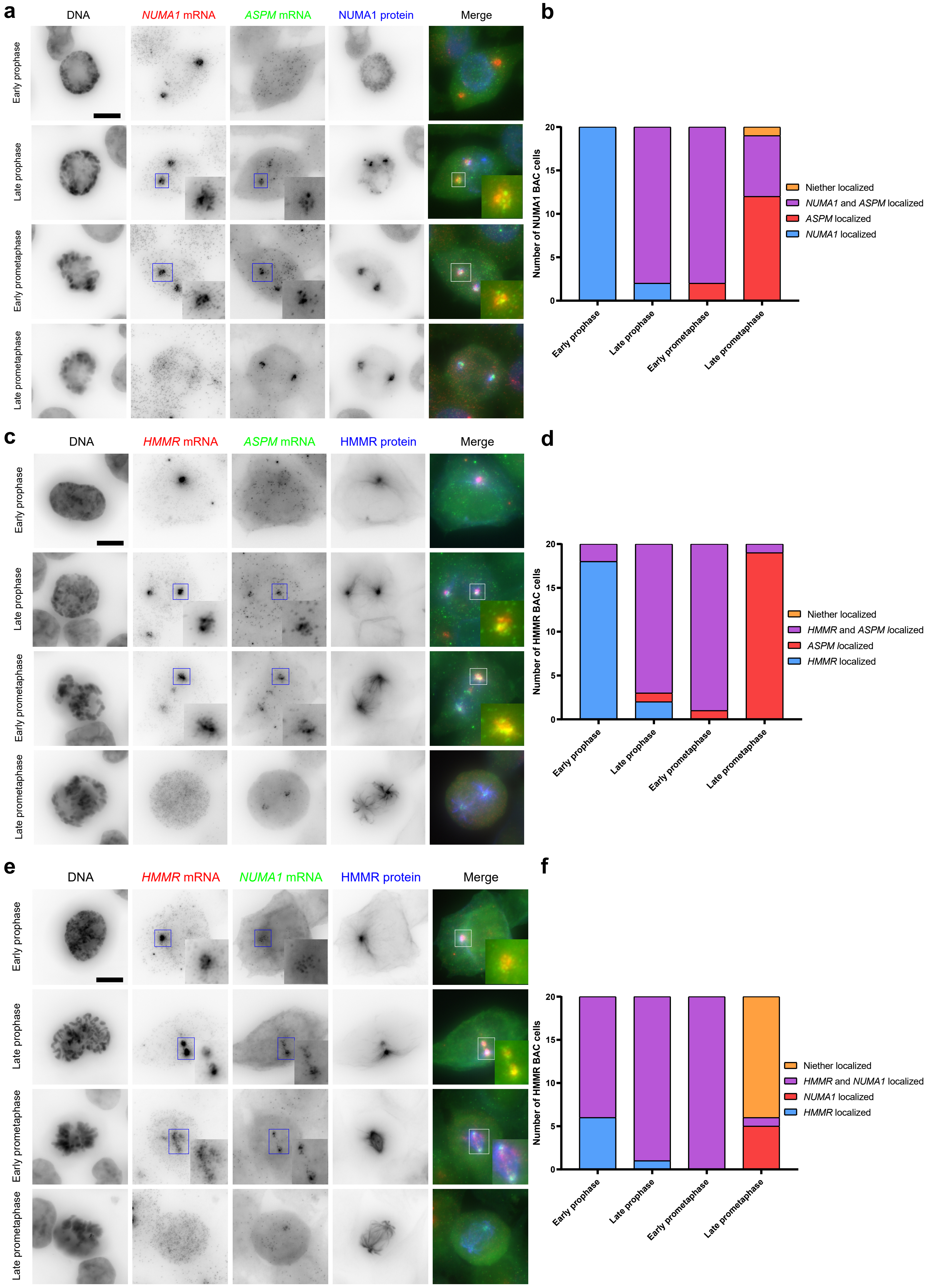
a
DNA
NUMA1 mRNA
ASPM mRNA
NUMA1 protein
Merge
Early prophase
Late prophase
Early prometaphase
Late prometaphase
b
Number of NUMA1 BAC cells
Niether localized
NUMA1 and ASPM localized
ASPM localized
NUMA1 localized
c
DNA
HMMR mRNA
ASPM mRNA
HMMR protein
Merge
d
Number of HMMR BAC cells
Niether localized
HMMR and ASPM localized
ASPM localized
HMMR localized
e
DNA
HMMR mRNA
NUMA1 mRNA
HMMR protein
Merge
f
Number of HMMR BAC cells
Niether localized
HMMR and NUMA1 localized
NUMA1 localized
HMMR localized

Figure S2

Supplementary Fig. 2 (related to Fig. 2): *ASPM, NUMA1,* and *HMMR* mRNAs localize to distinct peri-centrosomal regions and at precise times during early cell division.

a Images are micrographs of HeLa cells containing a NUMA1-GFP BAC and captured at different phases of early mitosis. Middle left and red: Cy3 fluorescent signals corresponding to *NUMA1-GFP* mRNAs labeled by smFISH; middle right and green: Cy5 signals corresponding to *ASPM* mRNA labeled by smiFISH. DNA stained with DAPI. Scale bar: 10 microns. Insets represent zooms of the boxed areas.

b Stacked histogram showing the number of cells having one, both, or neither transcript localized to centrosomes in each phase (n=20 cells per phase, counted from two independent experiments).

(**c**, **d**) Legend as in **a** and **b**, but for HeLa cells containing an HMMR-GFP BAC with *HMMR-GFP* mRNA labeled in Cy3 shown in red, and *ASPM* mRNA labeled in Cy5 and shown in green.

(**e**, **f**) Legend as in **a** and **b**, but for HeLa cells containing an HMMR-GFP BAC with *HMMR-GFP* mRNA labeled in Cy3 shown in red, and *NUMA1* mRNA labeled in Cy5 and shown in green.

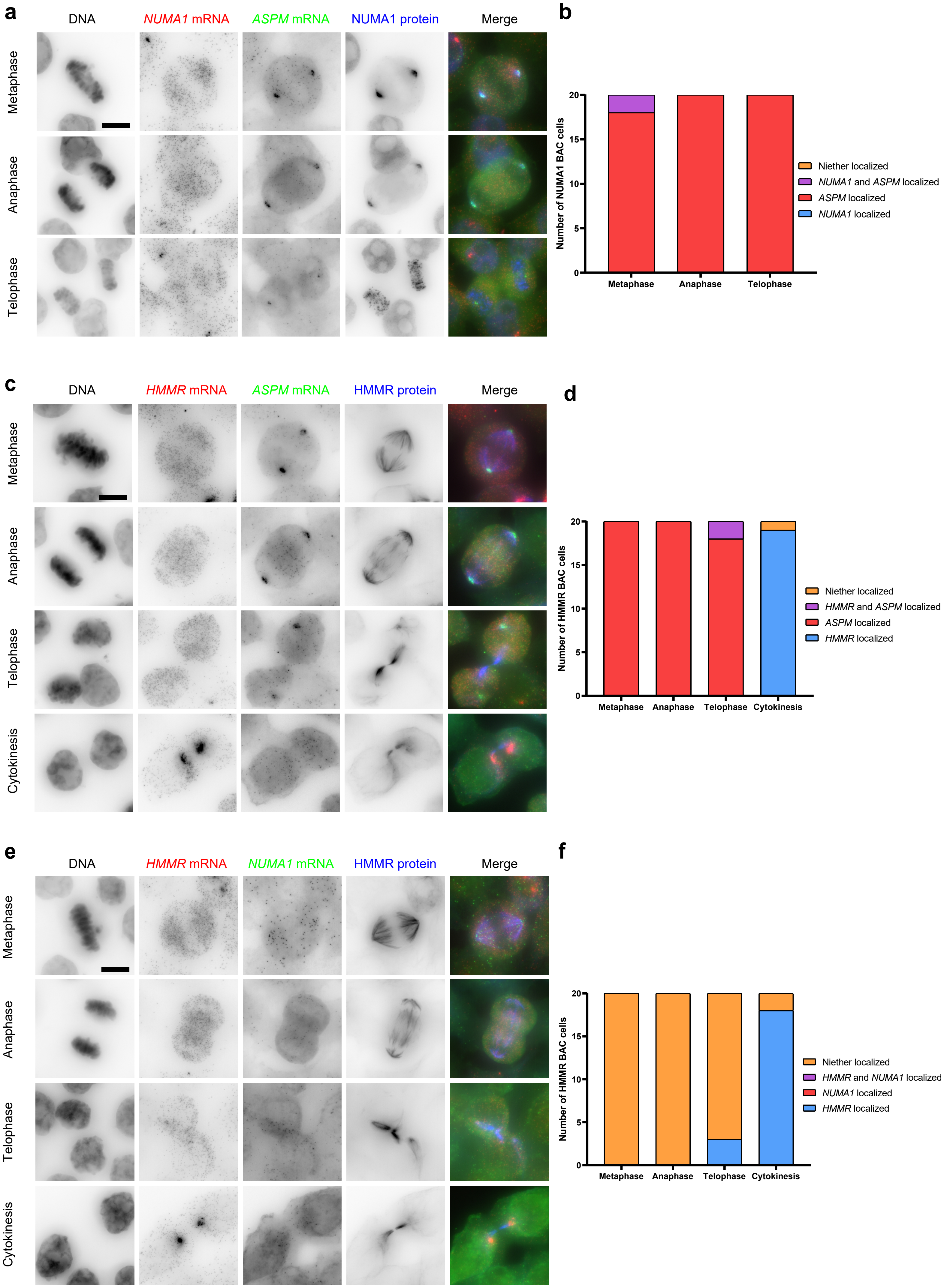

a
DNA
NUMA1 mRNA
ASPM mRNA
NUMA1 protein
Merge
Metaphase
Anaphase
Telophase
b
Number of NUMA1 BAC cells
0
5
10
15
20
Metaphase
Anaphase
Telophase
Niether localized
NUMA1 and ASPM localized
ASPM localized
NUMA1 localized
c
DNA
HMMR mRNA
ASPM mRNA
HMMR protein
Merge
Metaphase
Anaphase
Telophase
Cytokinesis
d
Number of HMMR BAC cells
0
5
10
15
20
Metaphase
Anaphase
Telophase
Cytokinesis
Niether localized
HMMR and ASPM localized
ASPM localized
HMMR localized
e
DNA
HMMR mRNA
NUMA1 mRNA
HMMR protein
Merge
Metaphase
Anaphase
Telophase
Cytokinesis
f
Number of HMMR BAC cells
0
5
10
15
20
Metaphase
Anaphase
Telophase
Cytokinesis
Niether localized
HMMR and NUMA1 localized
NUMA1 localized
HMMR localized

Figure S3

Supplementary Fig. 3 (related to Fig. 2): *ASPM, NUMA1,* and *HMMR* mRNAs differentially localize to centrosomes during late cell division.

a Images are micrographs of HeLa cells containing a NUMA1-GFP BAC and captured at different phases of late mitosis. Middle left and red: Cy3 fluorescent signals corresponding to *NUMA1* mRNAs labeled by smFISH; middle right and green: Cy5 signals corresponding to *ASPM* mRNA labeled by smiFISH. DNA stained with DAPI. Scale bar: 10 microns.

b Stacked histogram showing the number of cells having one, both, or neither transcript localized to centrosomes in each phase (n=20 cells per phase, counted from two independent experiments).

(**c**, **d**) Legend as in **a** and **b**, but for HeLa cells containing an HMMR-GFP BAC with *HMMR* mRNA labeled in Cy3 shown in red, and ASPM mRNA labeled in Cy5 and shown in green.

(**e**, **f**) Legend as in **a** and **b**, but for HeLa cells containing an HMMR-GFP BAC with *HMMR* mRNA labeled in Cy3 shown in red, and *NUMA1* mRNA labeled in Cy5 and shown in green.

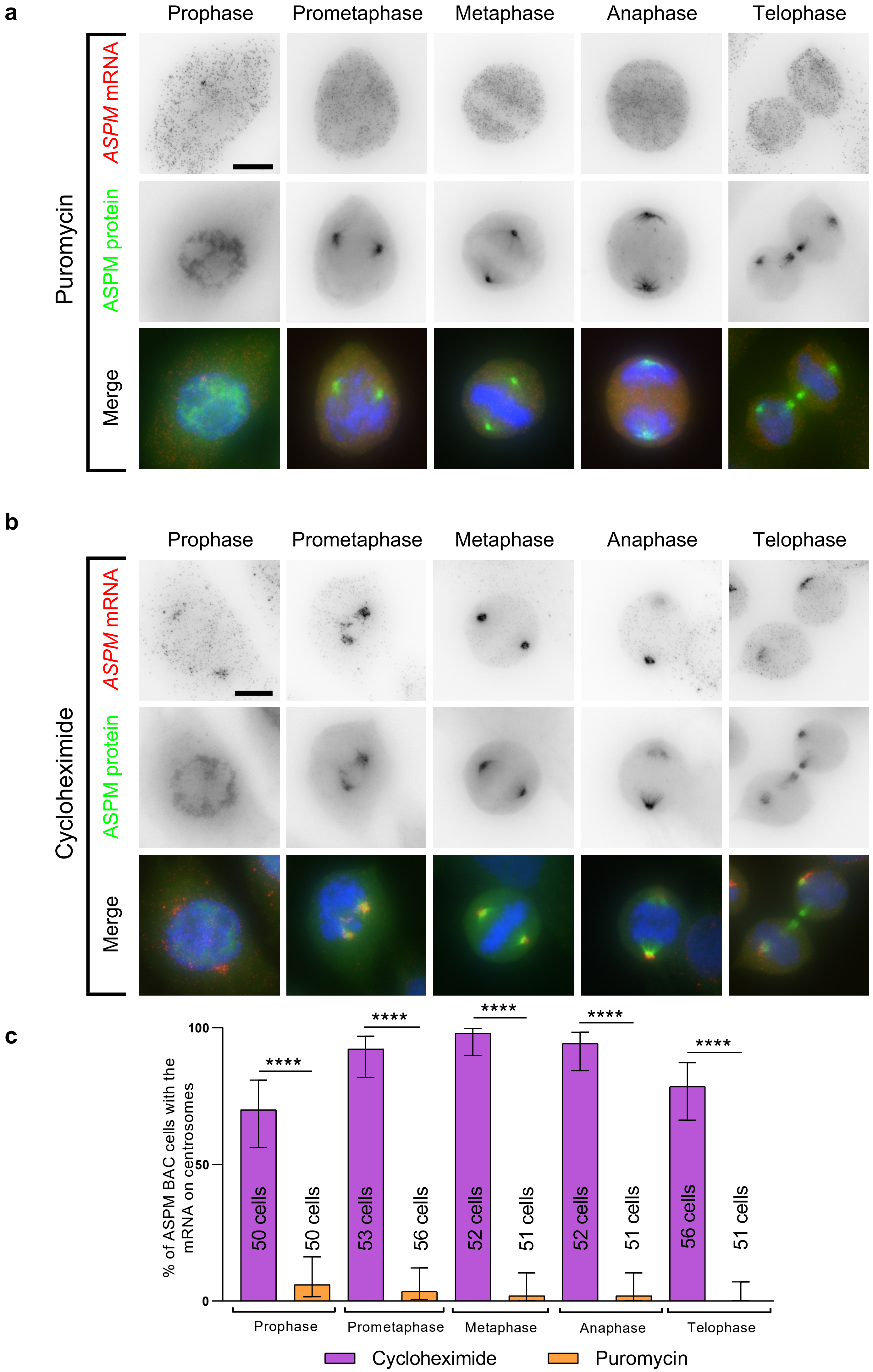
a
Prophase
Prometaphase
Metaphase
Anaphase
Telophase
Puromycin
ASPM mRNA
ASPM protein
Merge
b
Prophase
Prometaphase
Metaphase
Anaphase
Telophase
Cycloheximide
ASPM mRNA
ASPM protein
Merge
c
% of ASPM BAC cells with the mRNA on centrosomes
100
50
0
****
50 cells
50 cells
53 cells
56 cells
52 cells
51 cells
52 cells
51 cells
56 cells
51 cells
Prophase
Prometaphase
Metaphase
Anaphase
Telophase
Cycloheximide
Puromycin

Figure S4

Supplementary Fig. 4 (related to Fig. 3): Translation initiation is required for the localization of *ASPM* mRNAs during all phases of mitosis.

a Micrographs of HeLa cells expressing an ASPM-GFP BAC, treated with puromycin and imaged during mitosis. Up and red: Cy3 fluorescent signals corresponding to *ASPM* mRNAs labeled by smFISH with probes against the GFP RNA sequence; middle and green: GFP signals corresponding to the ASPM protein. Blue: DNA stained with DAPI. Scale bar: 10 microns.

b Legend same as in **a**, but for cells treated with cycloheximide.

c Bar graph depicting the percentage of cells showing a centrosomal localization of *ASPM-GFP* mRNAs after the indicated treatment. Data were analyzed from the total number of cells indicated in the bars from three independent experiments and expressed as a percentage of cells with localized mRNA. Binomial proportion 95% confidence intervals are shown in each case and were calculated using the Wilson/Brown method. Statistical significance was evaluated using a two-sided Fisher's exact test. **** indicates a p-value of <0.0001.

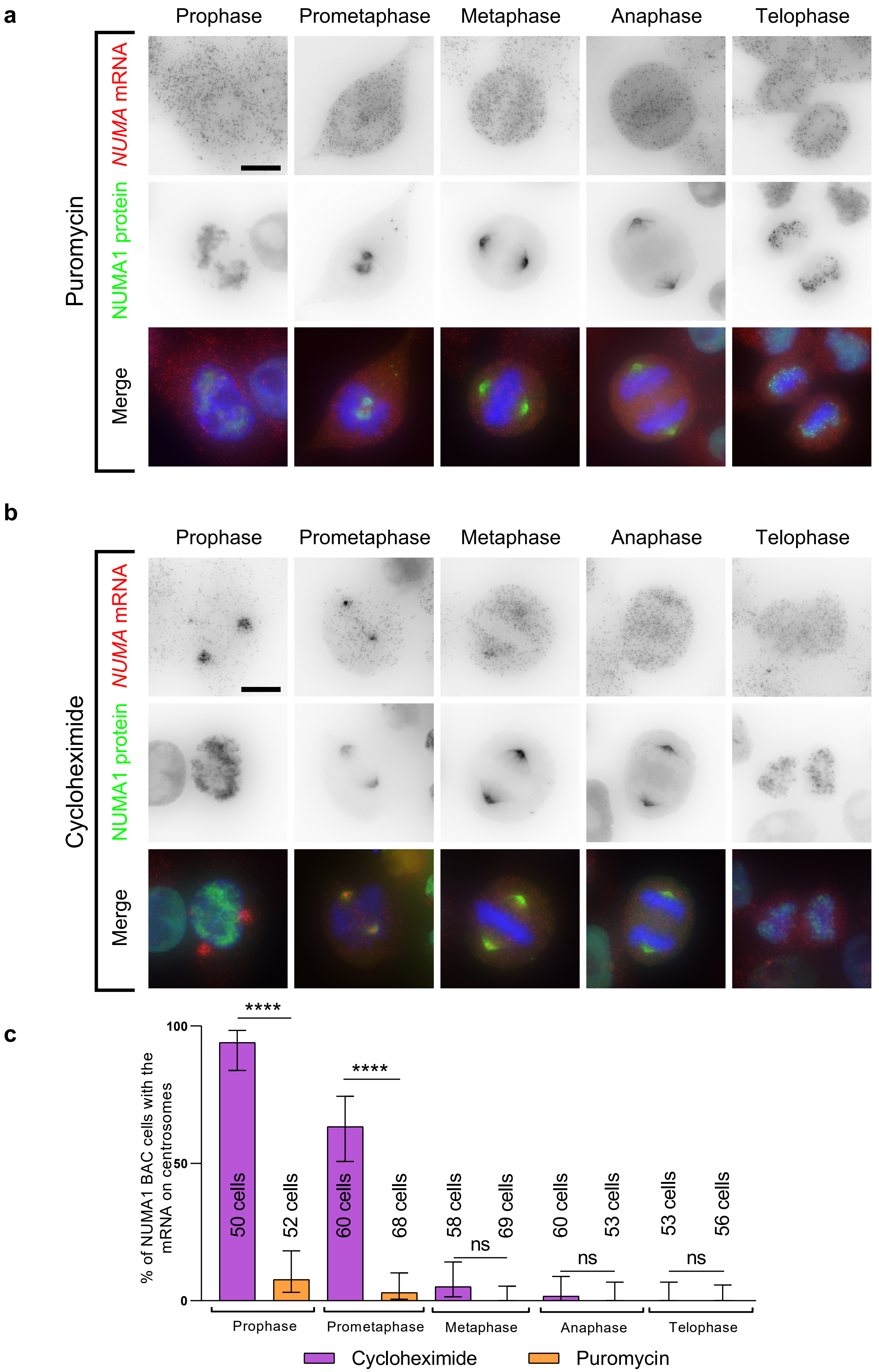

a
Prophase
Prometaphase
Metaphase
Anaphase
Telophase
Puromycin
NUMA mRNA
NUMA1 protein
Merge
b
Prophase
Prometaphase
Metaphase
Anaphase
Telophase
Cycloheximide
NUMA mRNA
NUMA1 protein
Merge
c
% of NUMA1 BAC cells with the mRNA on centrosomes
100
50
0
****
****
ns
ns
ns
50 cells
52 cells
60 cells
68 cells
58 cells
69 cells
60 cells
53 cells
53 cells
56 cells
Prophase
Prometaphase
Metaphase
Anaphase
Telophase
Cycloheximide
Puromycin

Figure S5

Supplementary Fig. 5 (related to Fig. 3): Translation initiation is required for the localization of *NUMA1* mRNAs during all phases of mitosis.

a Micrographs of HeLa cells expressing a NUMA1-GFP BAC, treated with puromycin and imaged during all phases of mitosis. Up and red: Cy3 fluorescent signals corresponding to *NUMA1-GFP* mRNA labeled by smFISH with probes against the GFP RNA sequence; middle and green: fluorescent signals corresponding to the NUMA1-GFP protein. Blue: DNA stained with DAPI. Scale bar: 10 microns.

b Legend same as in **a**, but for cells treated with cycloheximide.

c Bar graph depicting the percentage of cells showing a centrosomal localization of *NUMA1-GFP* mRNAs after the indicated treatment, Data were analyzed from the total number of cells indicated in the bars from three independent experiments and expressed as a percentage of cells with localized mRNA. Binomial proportion 95% confidence intervals are shown in each case and were calculated using the Wilson/Brown method. Statistical significance was evaluated using a two-sided Fisher's exact test. **** indicates a p-value of <0.0001, ns: not significant.

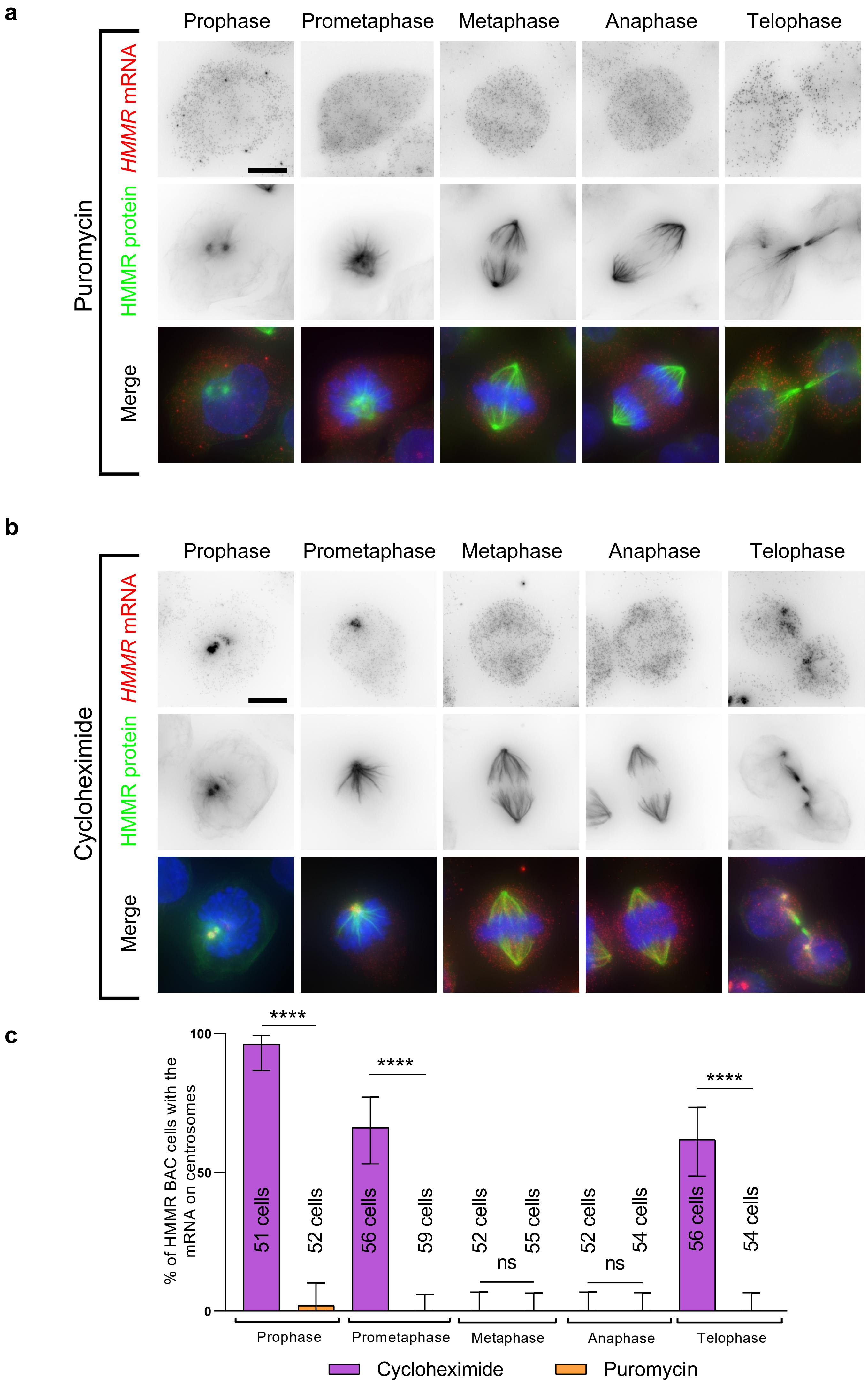

a
Prophase
Prometaphase
Metaphase
Anaphase
Telophase
Puromycin
HMMR mRNA
HMMR protein
Merge
b
Prophase
Prometaphase
Metaphase
Anaphase
Telophase
Cycloheximide
HMMR mRNA
HMMR protein
Merge
c
% of HMMR BAC cells with the mRNA on centrosomes
100
50
0
****
****
****
ns
ns
51 cells
52 cells
56 cells
59 cells
52 cells
55 cells
52 cells
54 cells
56 cells
54 cells
Prophase
Prometaphase
Metaphase
Anaphase
Telophase
Cycloheximide
Puromycin

Figure S6

Supplementary Fig. 6 (related to Fig. 3): Translation initiation is required for the localization of *HMMR* mRNAs during all phases of mitosis.

a Micrographs of HeLa cells expressing a HMMR-GFP BAC, treated with puromycin and imaged during mitosis. Up and red: Cy3 fluorescent signals corresponding to *HMMR-GFP* mRNAs labeled by smFISH with probes against the GFP RNA sequence; middle and green: fluorescent signals corresponding to the HMMR-GFP protein. Blue: DNA stained with DAPI. Scale bar: 10 microns.

b Legend same as in **a**, but for cells treated with cycloheximide.

c Bar graph depicting the percentage of cells showing a centrosomal localization of *HMMR-GFP* mRNAs after the indicated treatment. Data were analyzed from the total number of cells indicated in the bars from three independent experiments and expressed as a percentage of cells with localized mRNA. Binomial proportion 95% confidence intervals are shown in each case and were calculated using the Wilson/Brown method. Statistical significance was evaluated using a two-sided Fisher's exact test. **** indicates a p-value of <0.0001, ns: not significant.

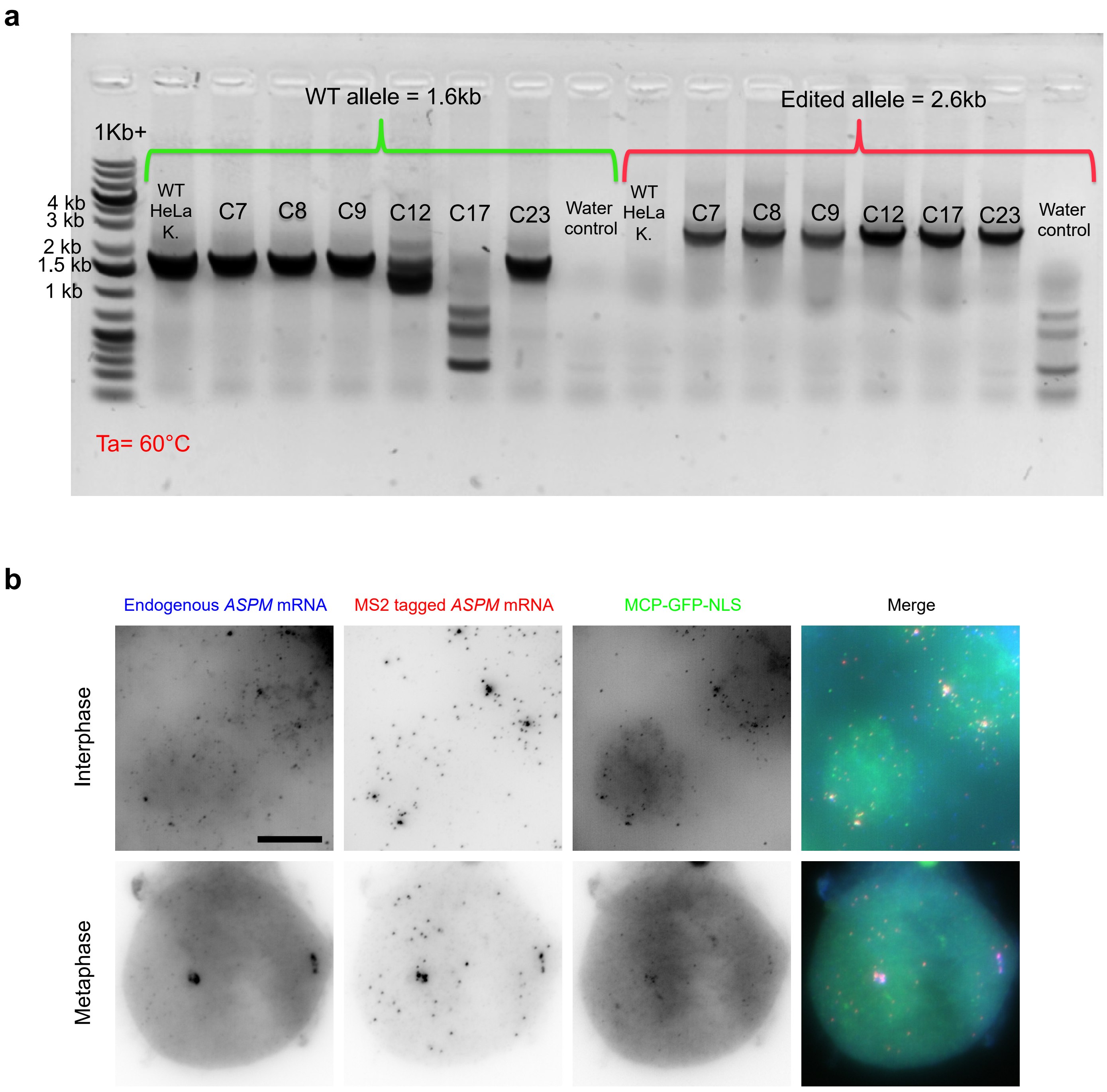
a
WT allele = 1.6kb
Edited allele = 2.6kb
1Kb+
4 kb
3 kb
2 kb
1.5 kb
1 kb
WT HeLa K.
C7
C8
C9
C12
C17
C23
Water control
WT HeLa K.
C7
C8
C9
C12
C17
C23
Water control
Ta= 60°C
b
Endogenous ASPM mRNA
MS2 tagged ASPM mRNA
MCP-GFP-NLS
Merge
Interphase
Metaphase

Figure S7

Supplementary Fig. 7 (related to Fig. 5): Characterization of the *ASPM-MS2x24* clone.

a Image is a scan of a gel loaded with the product of a PCR performed on genomic DNA extracted from various *ASPM-MS2x24* CRISPR clones. Wild-type and edited alleles are differentially amplified and give a product size of 1.6 and 2.6 kb, respectively. A ladder is placed on the left with the corresponding size markers. WT HeLa K.: PCR performed on the parental unedited cell line. Water control: PCR performed without any DNA. Ta: annealing temperature. PCR was performed once.

b Micrographs of *ASPM-MS2x24* HeLa cells expressing MCP-GFP-NLS and imaged at interphase and mitosis. Left and blue: Cy5 fluorescent signals corresponding to tagged and untagged *ASPM* mRNAs labeled by smiFISH with probes against the endogenous mRNA; middle and red: Cy3 signals corresponding to tagged mRNAs labeled by smFISH with probes against the MS2 sequence; right and green: GFP signals corresponding to *ASPM-MS2x24* mRNAs labeled by the MCP-GFP-NLS. Blue: DNA stained with DAPI. Scale bar: 10 microns. Experiment was performed twice with similar results.

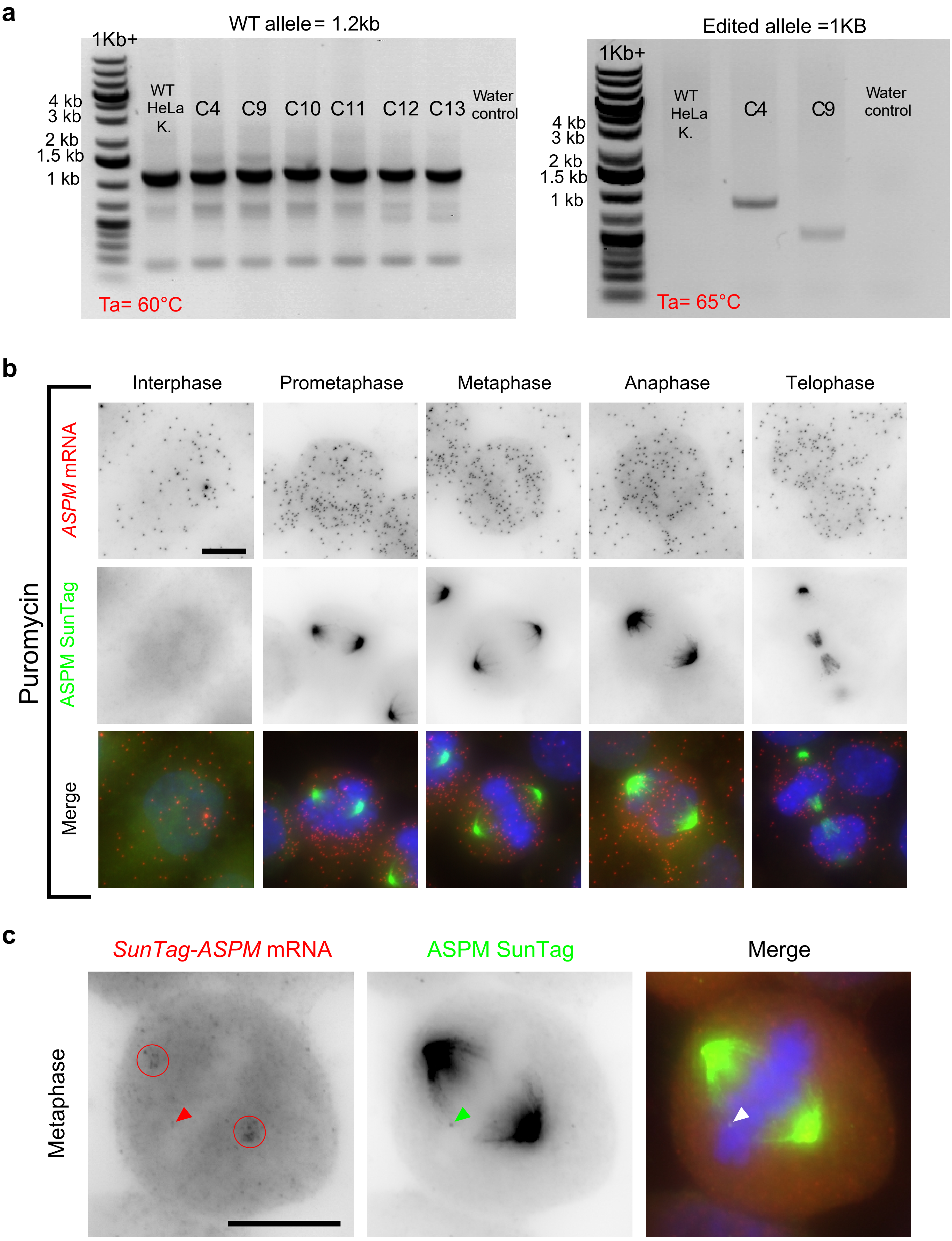
a
WT allele = 1.2kb
1Kb+
4 kb
3 kb
2 kb
1.5 kb
1 kb
WT HeLa K.
C4
C9
C10
C11
C12
C13
Water control
Ta= 60°C
Edited allele =1KB
1Kb+
4 kb
3 kb
2 kb
1.5 kb
1 kb
WT HeLa K.
C4
C9
Water control
Ta= 65°C
b
Interphase
Prometaphase
Metaphase
Anaphase
Telophase
Puromycin
ASPM mRNA
ASPM SunTag
Merge
c
SunTag-ASPM mRNA
ASPM SunTag
Merge
Metaphase

Figure S8

Supplementary Fig. 8 (related to Fig. 6): Characterization of the *SunTagx32-ASPM* clone.

a Image is a scan of a gel loaded with the product of a PCR performed on genomic DNA extracted from various *SunTagx32-ASPM* clones. Wild-type and edited alleles are differentially amplified and give a product size of 1.2 and 1 kb, respectively. A ladder is placed on the left with the corresponding size markers. WT HeLa K.: PCR performed on the parental unedited cell line. Water control: PCR performed without any DNA. Ta: annealing temperature. PCR was performed once.

b Micrographs of *SunTagx32-ASPM* HeLa cells expressing the scFv-sfGFP, treated with puromycin and imaged during interphase and mitosis. Upper and red: Cy3 fluorescent signals corresponding to tagged and untagged *ASPM* mRNAs labeled by smFISH; middle and green: GFP signals corresponding to the SunTagx32-ASPM mature protein. Blue: DNA stained with DAPI. Scale bar: 10 microns. Experiment was performed twice with similar results.

c Images are micrographs of a HeLa clone expressing endogenous *SunTagx32-ASPM* and scFv-sfGFP. Left and red: Cy3 fluorescent signals corresponding to *ASPM* mRNA tagged with 32 SunTag repeats revealed by smFISH against the SunTag and puromycin sequences; middle and green: GFP signals corresponding to the ASPM mature protein. Blue: DNA stained with DAPI. Scale bar: 10 microns. The red and green arrows indicate an mRNA and a polysome respectively. The red circles indicate clusters of tagged *ASPM* mRNA. Experiment was performed twice with similar results.

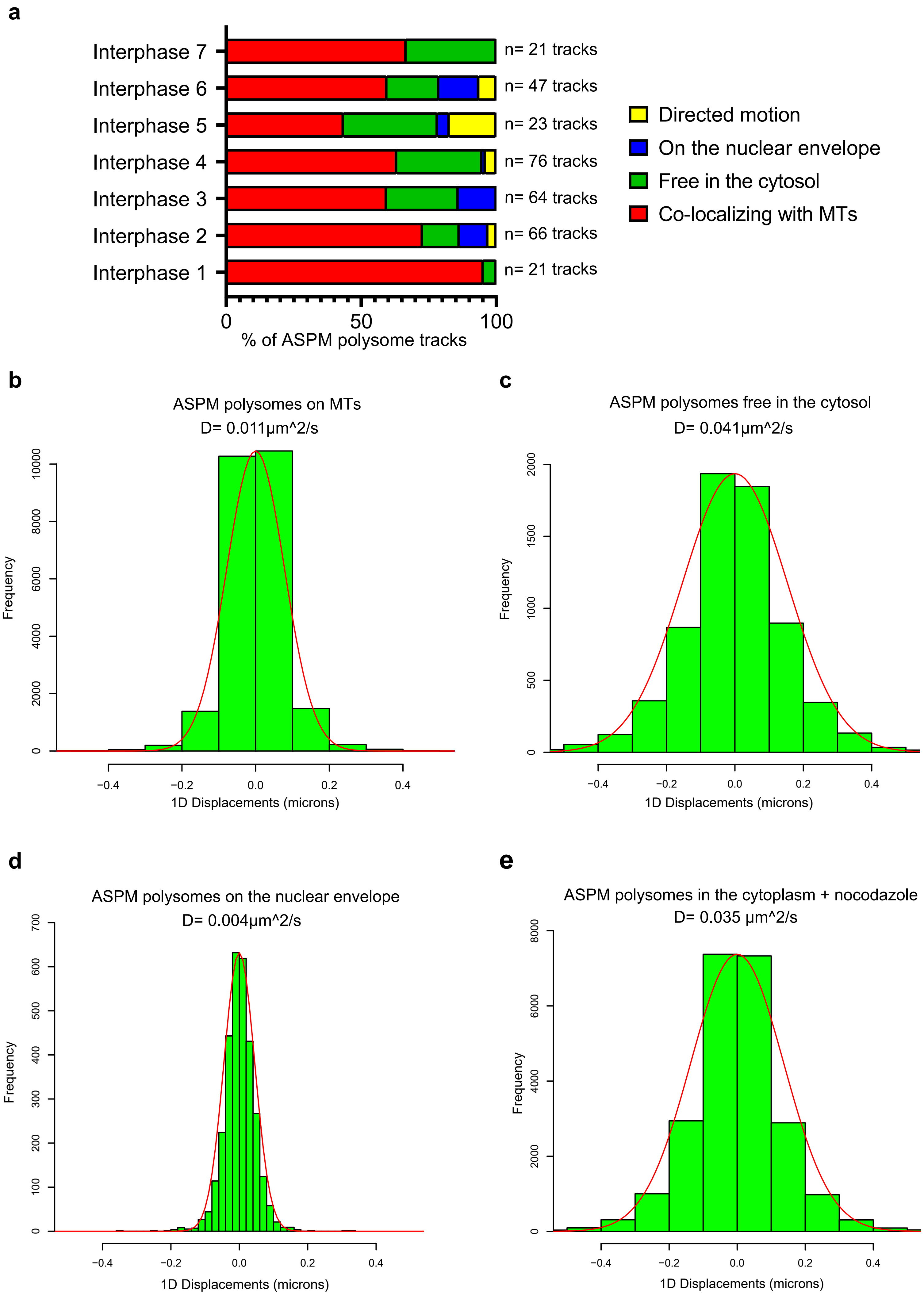

a
Interphase 7
Interphase 6
Interphase 5
Interphase 4
Interphase 3
Interphase 2
Interphase 1
n= 21 tracks
n= 47 tracks
n= 23 tracks
n= 76 tracks
n= 64 tracks
n= 66 tracks
n= 21 tracks
Directed motion
On the nuclear envelope
Free in the cytosol
Co-localizing with MTs
0
50
100
% of ASPM polysome tracks
b
ASPM polysomes on MTs
D= 0.011µm^2/s
Frequency
1D Displacements (microns)
c
ASPM polysomes free in the cytosol
D= 0.041µm^2/s
Frequency
1D Displacements (microns)
d
ASPM polysomes on the nuclear envelope
D= 0.004µm^2/s
Frequency
1D Displacements (microns)
e
ASPM polysomes in the cytoplasm + nocodazole
D= 0.035 µm^2/s
Frequency
1D Displacements (microns)

Supplementary Fig. 9 (related to Fig. 7): 1D displacements of ASPM polysomes.

a Stacked bar graphs showing the proportion of the ASPM polysome tracks in the indicated categories.

b Histogram showing 1D displacements measured between two consecutive time frames of ASPM polysome, for tracks localizing on MTs. D is the average diffusion coefficient. Data obtained across two independent experiments.

c Legend same as in **b**, but for ASPM polysome tracks free in the cytosol (neither on MTs nor on the nuclear envelope). Data obtained across two independent experiments.

d Legend same as in **b**, but for ASPM polysome tracks on the nuclear envelope.

e Legend same as in **b**, but for cells treated with nocodazole and ASPM polysomes not localizing at the nuclear envelope. Data obtained across two independent experiments.

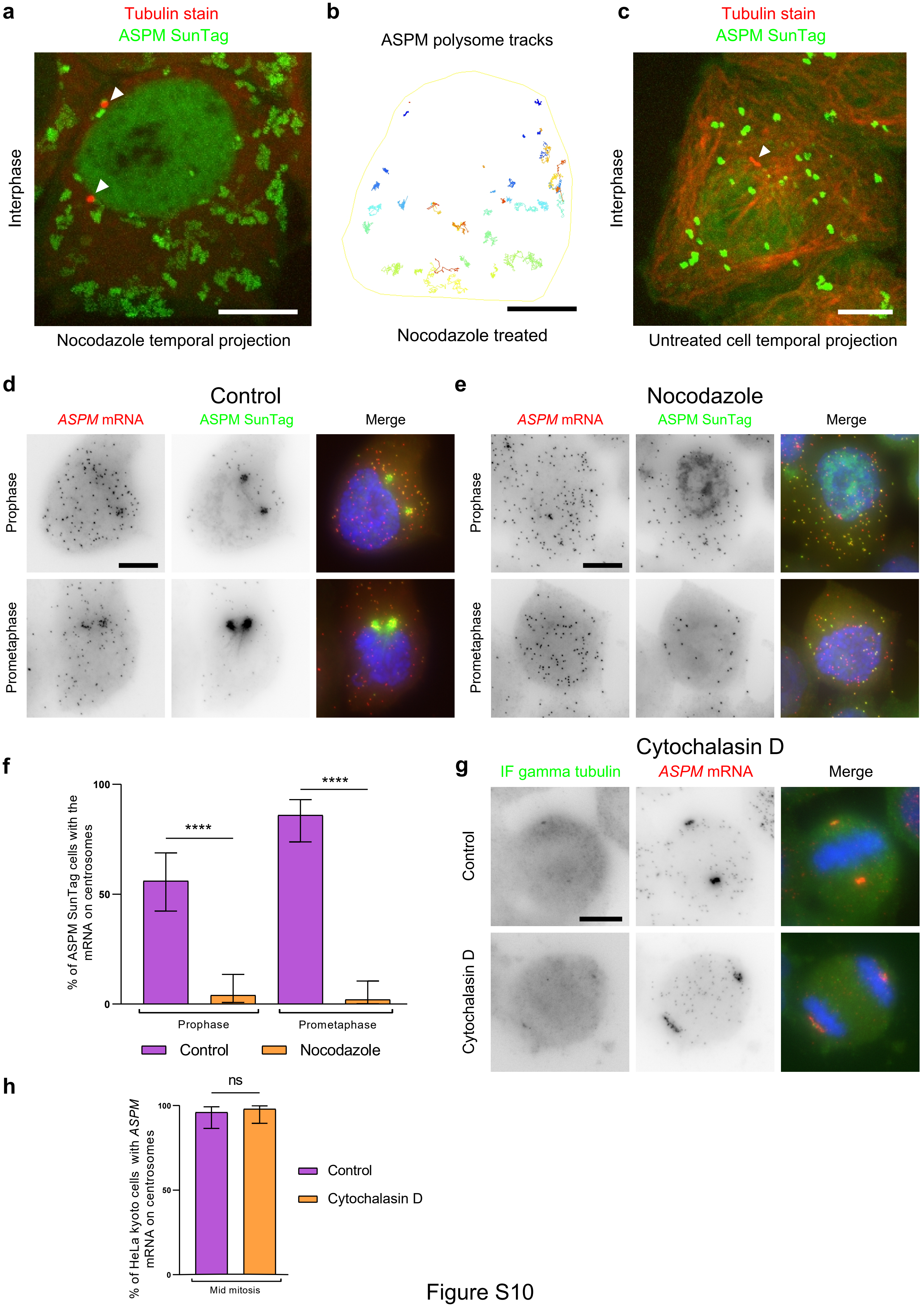
a
Tubulin stain
ASPM SunTag
Interphase
Nocodazole temporal projection
b
ASPM polysome tracks
Nocodazole treated
c
Tubulin stain
ASPM SunTag
Interphase
Untreated cell temporal projection
d
Control
ASPM mRNA
ASPM SunTag
Merge
Prophase
Prometaphase
e
Nocodazole
ASPM mRNA
ASPM SunTag
Merge
Prophase
Prometaphase
f
% of ASPM SunTag cells with the mRNA on centrosomes
100
50
0
****
****
Prophase
Prometaphase
Control
Nocodazole
g
Cytochalasin D
IF gamma tubulin
ASPM mRNA
Merge
Control
Cytochalasin D
h
% of HeLa kyoto cells with ASPM mRNA on centrosomes
ns
100
50
0
Mid mitosis
Control
Cytochalasin D

Figure S10

Supplementary Fig. 10 (related to Fig. 7): The effects of nocodazole on ASPM polysome dynamics and *ASPM* mRNA localization.

a Micrograph represents a temporal projection of *SunTagx32-ASPM* cells expressing scFv-sfGFP and imaged live during interphase, with labeled MT. The SunTag signal is shown in green and corresponds to ASPM polysomes and mature proteins; the far-red signal is shown in red and corresponds to a tubulin staining. Scale bar: 10 microns. White arrowhead indicates centrosomes.

b A TrackMate overlay of the same cell as in A, showing polysomes tracks. Color code represents displacement (dark blue lowest, red highest). The outer yellow outline represents the cell border.

c Same legend as in A, but for a control cell untreated with nocodazole.

d Micrographs of *SunTagx32-ASPM* cells expressing scFv-sfGFP and imaged at early mitosis. Left and red: Cy3 fluorescent signals corresponding to tagged and untagged *ASPM* mRNAs labeled by smFISH with probes against the endogenous mRNA; middle and green: GFP signals corresponding to SunTagx32-ASPM polysomes and mature protein. Blue: DNA stained with DAPI. Scale bar: 10 microns.

e Same legend as **e**, but with cells treated with nocodazole.

f Bar graph depicting the percentage of cells showing a centrosomal localization of *ASPM* mRNAs in the ASPM-SunTag clone after the indicated treatment. Data were analyzed from 50 cells per condition from two independent experiments and expressed as a percentage of cells with localized mRNA. Binomial proportion 95% confidence intervals are shown in each case and were calculated using the Wilson/Brown method. Statistical significance was evaluated using a two-sided Fisher's exact test. **** indicates a p-value of <0.0001, ns: not significant.

g Micrographs of HeLa Kyoto cells imaged at mid mitosis with or without a cytochalasin D treatment . Left and green: Cy5 fluorescent signals corresponding to a gamma tubulin immunofluorescence. Middle and red: *ASPM* mRNAs labeled by smFISH with probes against the endogenous mRNA. Blue: DNA stained with DAPI. Scale bar: 10 microns.

h same legend as **f**, but with HeLa Kyoto cells treated with Cytochalasin D.

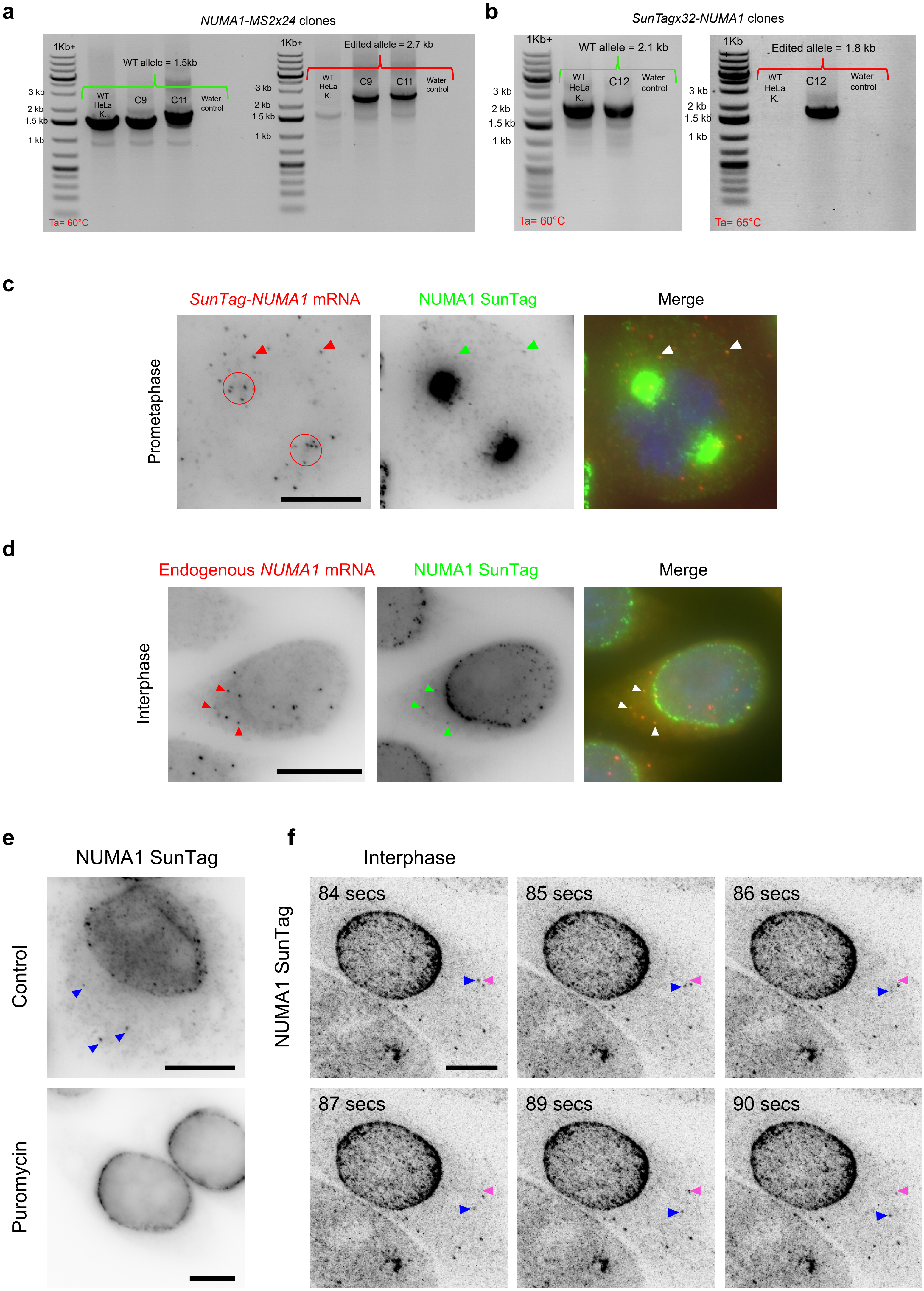
a
NUMA1-MS2x24 clones
1Kb+
WT allele = 1.5kb
3 kb
2 kb
1.5 kb
1 kb
WT HeLa K.
C9
C11
Water control
Edited allele = 2.7 kb
Ta= 60°C
b
SunTagx32-NUMA1 clones
WT allele = 2.1 kb
C12
Edited allele = 1.8 kb
1Kb
Ta= 65°C
c
SunTag-NUMA1 mRNA
NUMA1 SunTag
Merge
Prometaphase
d
Endogenous NUMA1 mRNA
Interphase
e
Control
Puromycin
f
84 secs
85 secs
86 secs
87 secs
89 secs
90 secs

Figure S11

Supplementary Fig. 11 (related to Fig. 8): Characterizing *NUMA1-MS2x24 and SunTagx32-NUMA1* clones.

a Image is a scan of a gel loaded with the product of a PCR performed on genomic DNA extracted from various *NUMA1-MS2x24* clones. Wild-type and edited alleles are differentially amplified and give a product size of 1.5 and 2.7 kbs respectively. A ladder is placed on the left of each amplification with the corresponding size markers. WT HeLa K.: PCR performed on the parental unedited cell line. Water control: PCR performed without any DNA. Ta: annealing temperature. PCR was performed once.

b Image is a scan of a gel loaded with the product of a PCR performed on genomic DNA extracted from a *SunTagx32-NUMA1* clone. Wild-type and edited alleles are differentially amplified and give a product size of 2.1 and 1.8 kbs respectively. A ladder is placed on the left of each amplification with the corresponding size markers. WT HeLa K.: PCR performed on the parental unedited cell line. Water control: PCR performed without any DNA. Ta: annealing temperature. PCR was performed once.

c Micrographs of a *SunTagx32-NUMA1* cells expressing the scFv-sfGFP. Left and red: Cy3 fluorescent signals corresponding to *SunTagx32-NUMA1* mRNAs, revealed by smFISH against the SunTag and puromycin sequences; middle and green: GFP signals corresponding to the SunTagx32-NUMA1 mature protein and polysomes. Blue: DNA stained with DAPI. Scale bar: 10 microns. The red and green arrows indicate an mRNA and a polysome respectively. The red circles indicate clusters of tagged NUMA1 mRNA. Experiment was performed twice with similar results.

d Micrographs of *SunTagx32-NUMA1* cells expressing the scFv-sfGFP and images during interphase. Left and red: Cy3 fluorescent signals corresponding to tagged and untagged *NUMA1* mRNAs labeled by smiFISH with probes against the endogenous mRNA; middle and green: GFP signals corresponding to SunTagx32-NUMA1

polysomes and mature proteins. Blue: DNA stained with DAPI. Scale bar: 10 microns. Red and green arrowheads indicate *SunTagx32-NUMA1* mRNAs and polysomes, respectively. White arrows indicate the overlay of red and green arrows. Experiment was performed twice with similar results.

e Micrographs of *SunTagx32-NUMA1* cells, with or without a puromycin treatment and imaged during interphase. The SunTag signal is shown in black. Blue arrowheads indicate polysomes. Scale bars represent 10 microns. Experiment was performed twice with similar results.

f Snapshots of a living *SunTagx32-NUMA1* cell imaged during interphase. The SunTag signal is shown in black and corresponds to ASPM polysomes and mature proteins. Scale bar: 10 microns. Time is in seconds. Pink arrowheads indicate the starting position of a polysome, while blue ones follow its position at the indicated time. Experiment was performed three times with similar results.

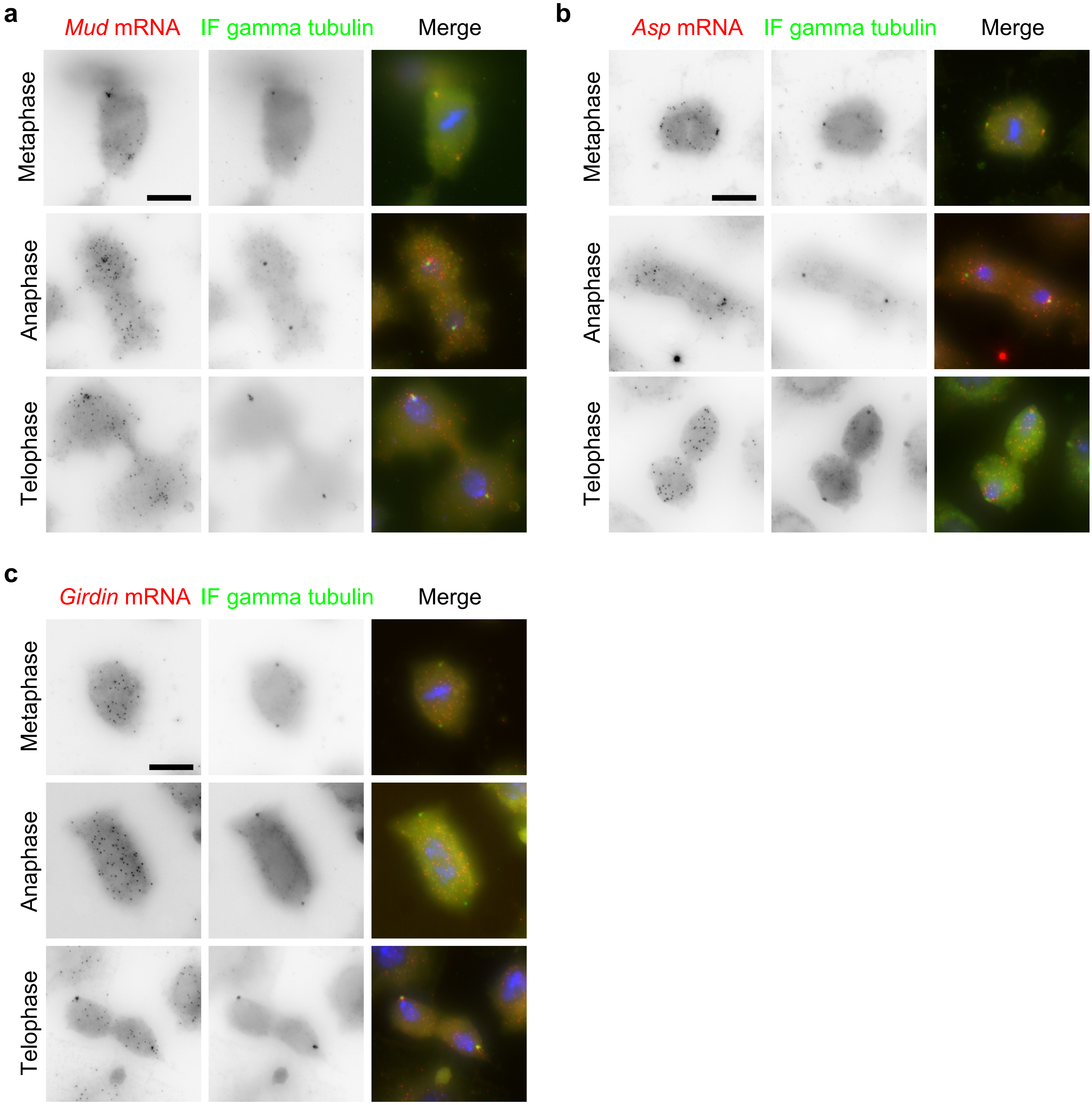
a
Mud mRNA
IF gamma tubulin
Merge
Metaphase
Anaphase
Telophase
b
Asp mRNA
IF gamma tubulin
Merge
Metaphase
Anaphase
Telophase
c
Girdin mRNA
IF gamma tubulin
Merge
Metaphase
Anaphase
Telophase

Figure S12

Supplementary Fig. 12 (related to Fig. 9): Cell cycle dependent centrosomal mRNA localization in S2R+ cells.

a Images are micrographs of S2R+ cells during metaphase, anaphase, and telophase. Left and red: Cy3 fluorescent signals corresponding to *Mud* mRNAs labeled by smiFISH; middle and green: fluorescent signals corresponding to the gamma tubulin protein revealed by IF. Blue: DNA stained with DAPI. Scale bar: 10 microns. Experiment was performed three times with similar results.

b Legend as in **a**, but for *Asp* mRNA.

c Legend as in **a**, but for *Girdin* mRNA.

Supplementary table 1: primers used for genotyping

Primer name	Primer sequence
ASPM gene end WT forward	5’-TCAGAGGGTATGGAGGGGAA-3’
ASPM gene end WT reverse	5’-GACATCTGTGGCCCTGAAAC-3’
ASPM gene end forward	5’-TCAGAGGGTATGGAGGGGAA-3’
IRES reverse	5’-GCCCTCACATTGCCAAAAGA-3’
NUMA1 gene end WT forward	5’-ACCAAGGACTAAAGGGAGCC-3’
NUMA1 gene end WT reverse	5’-CAACCCCACTCCTGAGACAT-3’
ASPM gene start WT forward	5’-TGTTCCTGGAAACCGCAATG-3’
ASPM gene start WT reverse	5’-GTTTATGTGTTGTCCCCGCC-3’
SunTagx32 forward	5’- AAAAGGGTAGCGGATCAGGA-3’
ASPM gene start reverse	5’- CATGTGTATGCGTCAAGGGC -3’
NUMA1 gene start WT forward	5’-TCATTGTGCCCCTGGAGATT-3’
NUMA1 gene start WT reverse	5’-CAGAGAGACCAGTGCTGTGA-3’
FLAG forward	5’-ACCGGTGACTACAAAGACGA-3’
NUMA1 gene start reverse	5’-GCTGTGATTCTATGCTGGGC-3’
