# Supplementary material for: A choreography of centrosomal mRNAs reveals a conserved localization mechanism involving active polysome transport
Source: Nat Commun. 2021 Mar 1;12:1352. doi: 10.1038/s41467-021-21585-7 (PMC7921559; doi:10.1038/s41467-021-21585-7)
Supplement: Supplementary file 15 — Reporting Summary [file 41467_2021_21585_MOESM15_ESM.pdf]

nature research

Corresponding author(s): Adham SAFIEDDINE
Edouard BERTRAND

Last updated by author(s): Dec 19, 2020

# Reporting Summary

Nature Research wishes to improve the reproducibility of the work that we publish. This form provides structure for consistency and transparency in reporting. For further information on Nature Research policies, see our Editorial Policies and the Editorial Policy Checklist.

## Statistics

For all statistical analyses, confirm that the following items are present in the figure legend, table legend, main text, or Methods section.

| n/a | Confirmed | |
|---|---|---|
| ☐ | ☒ | The exact sample size (*n*) for each experimental group/condition, given as a discrete number and unit of measurement |
| ☐ | ☒ | A statement on whether measurements were taken from distinct samples or whether the same sample was measured repeatedly |
| ☐ | ☒ | The statistical test(s) used AND whether they are one- or two-sided<br>*Only common tests should be described solely by name; describe more complex techniques in the Methods section.* |
| ☒ | ☐ | A description of all covariates tested |
| ☒ | ☐ | A description of any assumptions or corrections, such as tests of normality and adjustment for multiple comparisons |
| ☐ | ☒ | A full description of the statistical parameters including central tendency (e.g. means) or other basic estimates (e.g. regression coefficient) AND variation (e.g. standard deviation) or associated estimates of uncertainty (e.g. confidence intervals) |
| ☐ | ☒ | For null hypothesis testing, the test statistic (e.g. *F*, *t*, *r*) with confidence intervals, effect sizes, degrees of freedom and *P* value noted<br>*Give P values as exact values whenever suitable.* |
| ☒ | ☐ | For Bayesian analysis, information on the choice of priors and Markov chain Monte Carlo settings |
| ☒ | ☐ | For hierarchical and complex designs, identification of the appropriate level for tests and full reporting of outcomes |
| ☒ | ☐ | Estimates of effect sizes (e.g. Cohen's *d*, Pearson's *r*), indicating how they were calculated |

*Our web collection on statistics for biologists contains articles on many of the points above.*

## Software and code

Policy information about availability of computer code

| | |
|---|---|
| Data collection | Data collection: ImageJ 1.53d, Metamorph 7.8.8.0, Andor iQ3 3.6.3,<br>Figure generation: Adobe Photoshop 13.0.6 , Adobe Illustrator 23.0.3, OMERO 627737a5 |
| Data analysis | All the code used in data analysis as well as the generated raw numerical results can be fount at https://github.com/Henley13/paper_centrosome_2020 and http://dx.doi.org/10.5281/zenodo.4322750 |

For manuscripts utilizing custom algorithms or software that are central to the research but not yet described in published literature, software must be made available to editors and reviewers. We strongly encourage code deposition in a community repository (e.g. GitHub). See the Nature Research guidelines for submitting code & software for further information.

## Data

Policy information about availability of data

All manuscripts must include a data availability statement. This statement should provide the following information, where applicable:

- Accession codes, unique identifiers, or web links for publicly available datasets
- A list of figures that have associated raw data
- A description of any restrictions on data availability

Data availability statement included in the manuscript: Source data are provided with this paper. All relevant data that support the findings of this study are available from the corresponding authors upon reasonable request.

# Field-specific reporting

Please select the one below that is the best fit for your research. If you are not sure, read the appropriate sections before making your selection.

☒ Life sciences ☐ Behavioural & social sciences ☐ Ecological, evolutionary & environmental sciences

For a reference copy of the document with all sections, see nature.com/documents/nr-reporting-summary-flat.pdf

# Life sciences study design

All studies must disclose on these points even when the disclosure is negative.

| | |
|---|---|
| Sample size | In fixed samples, sample size was determined by counting single cells across different stages of the cell cycle. All counts were done across two or three independent experiments for each condition. During interphase, 100 cells were counted per condition. During mitosis, 20 to around 70 cells were counted in each phase per condition. When the cell cycle stage is not taken into account, no less than 50 cells were counted.<br>In living cells, cells were imaged during at least two independent experiments per condition.<br>For the automated quantification of centrosomal mRNA localization, more than 40,000 cells were imaged using an automated spinning disk confocal microscope.<br>These sample sizes were chosen based on the number of mitotic and interphase cells available in each imaging sample. They were sufficient to distingish mRNA localization patterns and the effects of drug treatments with adequate statistical tests. |
| Data exclusions | Based on visual inspection,we excluded the following cells: (i) cells that appear to be dying, (ii) cells that have unusually high background signal impeding single molecule detection (smFISH experiments).<br>In our live imaging experiments, we additionally excluded cells not expressing the mRNA/protein of interest (ASPM and NUMA1 mRNA and polysome live imaging experiments).<br>In the automated quantification of centrosomal mRNA localization, we excluded cells that show more than 2 centrosomes, and cells with less than 10 mRNA molecules detected (Fig. 3: smFISH in high-throughput experiment). |
| Replication | smFISH experiments, transient transfections, and live imaging experiments were repeated at least twice with success. Signals and expression levels/localisation patterns were very similar across replicates. For all other experiments, the number of repetitions is indicated in the figure legend. All attempts at replication were succesful. |
| Randomization | Experimental groups were assigned based on whether cells were treated with a specific drug inhibitor. |
| Blinding | Blinding was not relevant to this study since it based on cultured cells. All data is based on quantitative analyses. |

# Reporting for specific materials, systems and methods

We require information from authors about some types of materials, experimental systems and methods used in many studies. Here, indicate whether each material, system or method listed is relevant to your study. If you are not sure if a list item applies to your research, read the appropriate section before selecting a response.

## Materials & experimental systems

| n/a | Involved in the study | |
|---|---|---|
| ☐ | ☒ | Antibodies |
| ☐ | ☒ | Eukaryotic cell lines |
| ☒ | ☐ | Palaeontology and archaeology |
| ☒ | ☐ | Animals and other organisms |
| ☒ | ☐ | Human research participants |
| ☒ | ☐ | Clinical data |
| ☐ | ☒ | Dual use research of concern |

## Methods

| n/a | Involved in the study | |
|---|---|---|
| ☒ | ☐ | ChIP-seq |
| ☒ | ☐ | Flow cytometry |
| ☒ | ☐ | MRI-based neuroimaging |

# Antibodies

| | |
|---|---|
| Antibodies used | Monoclonal anti-γ-tubulin primary antibody produced in mouse (Sigma-Aldrich, T5326, clone GTU-88)<br>Polyclonal FITC-labeled anti-mouse secondary antibody produced in goat (Jackson ImmunoResearch 115-095-062)<br>Polyclonal Cy5-labeled anti-mouse secondary antibody produced in goat (Jackson ImmunoResearch 115-176-003) |
| Validation | Citations for the anti-γ-tubulin primary antibody include:<br>Association of Aurora A and gamma-tubulin expression in astrocytomas and patient survival. Tsai HP Neurological Research 36(8), 746-751, (2014)<br>Cilia localization is essential for in vivo functions of the Joubert syndrome protein Arl13b/Scorpion. Duldulao NA and S, Sun Z Development 136(23), 4033-4042, (2009)<br>BRCA1-dependent ubiquitination of gamma-tubulin regulates centrosome number. Starita LM Molecular and Cellular Biology 24(19), 8457-8466, (2004) |

# Eukaryotic cell lines

Policy information about cell lines

| | |
|---|---|
| Cell line source(s) | HeLa Kyoto cells stably expressing a BAC originated and were a gift from the lab of A. Hyman. Publication describing thier generation: Poser, I., Sarov, M., Hutchins, J. et al. BAC TransgeneOmics: a high-throughput method for exploration of protein function in mammals. Nat Methods 5, 409–415 (2008). https://doi.org/10.1038/nmeth.1199<br><br>HeLa cells expressing centrin 1-GFP were a gift from the lab of B. Delaval and originated from: Piel, M., Meyer, P., Khodjakov, A., Rieder, C. L. & Bornens, M. The Respective Contributions of the Mother and Daughter Centrioles to Centrosome Activity and Behavior in Vertebrate Cells. J. Cell Biol. 149, 317–330 (2000). https://doi.org/10.1083/jcb.149.2.317 |
| Authentication | All BAC expressing HeLa kyoto clones showed a GFP signal reflecting the tagged protein of interest. Similarly, HeLa centrin 1 GFP cells displayed typical Centrin 1 expression patterns. Furthermore, single molecule FISH experiments using specific probes recognising human and Drosophila transcripts revealed single molecules in HeLa and SR2R+ cells respectively, demonstrating they are human and drosophila cell lines. |
| Mycoplasma contamination | We confirm that all cell lines tested negative for Mycoplamsa. |
| Commonly misidentified lines<br>(See ICLAC register) | No commonly misidentified lines were used in this study. |

# Dual use research of concern

Policy information about dual use research of concern

## Hazards

Could the accidental, deliberate or reckless misuse of agents or technologies generated in the work, or the application of information presented in the manuscript, pose a threat to:

| No | Yes | |
|---|---|---|
| ☒ | ☐ | Public health |
| ☒ | ☐ | National security |
| ☒ | ☐ | Crops and/or livestock |
| ☒ | ☐ | Ecosystems |
| ☒ | ☐ | Any other significant area |

## Experiments of concern

Does the work involve any of these experiments of concern:

| No | Yes | |
|---|---|---|
| ☒ | ☐ | Demonstrate how to render a vaccine ineffective |
| ☒ | ☐ | Confer resistance to therapeutically useful antibiotics or antiviral agents |
| ☒ | ☐ | Enhance the virulence of a pathogen or render a nonpathogen virulent |
| ☒ | ☐ | Increase transmissibility of a pathogen |
| ☒ | ☐ | Alter the host range of a pathogen |
| ☒ | ☐ | Enable evasion of diagnostic/detection modalities |
| ☒ | ☐ | Enable the weaponization of a biological agent or toxin |
| ☒ | ☐ | Any other potentially harmful combination of experiments and agents |
