# Supplementary material for: A choreography of centrosomal mRNAs reveals a conserved localization mechanism involving active polysome transport
Source: Nat Commun. 2021 Mar 1;12:1352. doi: 10.1038/s41467-021-21585-7 (PMC7921559; doi:10.1038/s41467-021-21585-7)
Supplement: Supplementary file 16 — Description of Additional Supplementary Files [file 41467_2021_21585_MOESM16_ESM.docx]

Description of Additional Supplementary files

Title: Supplementary Movie 1

Description: ASPM mRNA anchored on centrosomes and showing directed movements during metaphase. HeLa cells expressing the edited ASPM-MS2x24 allele and MCP-GFP-NLS were imaged every 0.7 seconds for around 70 seconds during metaphase. The GFP signal shown in black corresponds to ASPM-MS2x24 mRNAs. The red arrow follows an mRNA showing directed movement.

Title: Supplementary Movie 2

Description: ASPM mRNA anchored on the mitotic spindle. HeLa cells expressing the edited ASPM-MS2x24 allele and MCP-GFP-NLS were imaged every 0.625 seconds for around 120 seconds during prometaphase. The GFP signal shown in green corresponds to ASPM- MS2x24 mRNAs; the Cy5 signal shown in red corresponds to DNA and was imaged for a single frame.

Title: Supplementary Movie 3

Description: ASPM polysome dynamics during prophase. : HeLa cells expressing the edited SunTagx32-ASPM allele and scFv-sfGFP were imaged every 0.9 seconds for 180 seconds during prophase. The GFP signal shown in black corresponds to ASPM polysomes and mature protein.

Title: Supplementary Movie 4

Description: ASPM polysome dynamics during prometaphase. HeLa cells expressing the edited SunTagx32-ASPM allele and scFv-sfGFP were imaged every 0.9 seconds for 180 seconds during prometaphase. The GFP signal shown in black corresponds to ASPM polysomes and mature protein.

Title: Supplementary Movie 5

Description: ASPM polysome and microtubule dynamics during interphase. HeLa cells expressing the edited SunTagx32-ASPM allele and scFv-sfGFP were imaged every 0.66 seconds for 130 seconds during interphase with labeled microtubules. The GFP signal shown in green corresponds to ASPM polysomes and mature protein; the far-red signal shown in red corresponds to microtubules.

Title: Supplementary Movie 6

Description: ASPM polysome dynamics in nocodazole-treated interphase cells. HeLa cells treated with nocodazole, expressing the edited SunTagx32-ASPM allele and scFv-sfGFP were imaged every 0.53 seconds for 105 seconds during interphase with labeled MTs. The GFP signal shown in green corresponds to ASPM polysomes and mature protein; the far-red signal shown in red corresponds to microtubules and centrosomes.

Title: Supplementary Movie 7

Description: ASPM polysomes sliding on microtubules during prophase. HeLa cells expressing the edited SunTagx32-ASPM allele and scFvsfGFP were imaged every 0.66 seconds for 260 seconds during prophase with labeled microtubules. The GFP signal shown in green corresponds to ASPM polysomes and mature protein; the far-red signal shown in red corresponds to microtubules.

Title: Supplementary Movie 8

Description: ASPM polysomes pulled by microtubules during prometaphase. HeLa cells expressing the edited SunTagx32-ASPM allele and scFv-sfGFP were imaged every 0.66 seconds for 130 seconds during prometaphase with labeled MTs. The GFP signal shown in green corresponds to ASPM polysomes and mature protein; the far-red signal shown in red corresponds to microtubules.

Title: Supplementary Movie 9

Description: NUMA1 mRNA dynamics during prometaphase. HeLa cells expressing the edited NUMA1-MS2x24 allele and MCP-GFP-NLS were imaged every 0.9 seconds for around 80 seconds during prometaphase. The GFP signal shown in black corresponds to NUMA1 mRNA. The red arrow follows an mRNA showing directed movement.

Title: Supplementary Movie 10

Description: NUMA1 polysome dynamics during prometaphase. HeLa cells expressing the edited SunTagx32-NUMA1 allele and scFv-sfGFP were imaged every 0.74 seconds for around 180 seconds during prometaphase. The GFP signal shown in black corresponds to NUMA1 polysomes and mature protein.

Title: Supplementary Data 1

Description: Summary of all screened mRNAs.

Title: Supplementary Data 2

Description: Sequence of all smFISH and smiFISH probes.
